# Is Loneliness After Widowhood Less Prevalent in Recent Times Than Decades Ago?

**DOI:** 10.1093/geroni/igaa057.2029

**Published:** 2020-12-16

**Authors:** Bianca Suanet, Theo van Tilburg

**Affiliations:** 1 Vrije Universiteit Amsterdam, Amsterdam, Noord-Holland, Netherlands; 2 Vrije Universiteit, Amsterdam, Noord-Holland, Netherlands

## Abstract

Due to increased longevity, widowhood occurs much later in life. When comparing over decades, we hypothesize that the increase in loneliness after losing the spouse at an early age is currently higher due to the lack of role models and knowledge of what helps in this situation, and that the recovery of loneliness after widowhood is faster because the widowed get more network attention in that exceptional situation. We analyze data from the Longitudinal Aging Study Amsterdam on 232 widowers and 468 widows with an average of four observations before and four after widowhood, spread over 21 years. The younger and recent widowed had a greater increase in loneliness, but also better recovery than the older widowed and those widowed years ago. The loneliness of widowed people has decreased regardless of age at the event, indicating the greater potential for bouncing back from this life event in today’s society.

